# Predicting the severity of COVID-19 patients using the CD24-CSF1R index in whole blood samples

**DOI:** 10.1016/j.heliyon.2023.e13945

**Published:** 2023-02-23

**Authors:** Dat Nguyen Thanh, Nguyen Tan Thanh Giang, Tam Vy Le, Ngoc Minh Truong, Thanh Van Ngo, Thien Ngoc Lam, Dinh Truong Nguyen, Quynh Hoa Tran, Minh Nam Nguyen

**Affiliations:** a School of Medicine, Vietnam National University Ho Chi Minh City, Ho Chi Minh City 700000, Viet Nam; b Research Center for Genetics and Reproductive Health, School of Medicine, Vietnam National University Ho Chi Minh City, Ho Chi Minh City 700000, Viet Nam; c School of Biotechnology, Tan Tao University, Duc Hoa, Tan Duc E.City, Long An, 850000, Viet Nam; d Department of Biotechnology, International University - Vietnam National University Ho Chi Minh City, Ho Chi Minh City 700000, Viet Nam; e Department of Pathology, Tulane University School of Medicine, Tulane Cancer Center, New Orleans, LA, United States; f Department of Biology and Environment, Ho Chi Minh City University of Food Industry (HUFI), Ho Chi Minh City 700000, Viet Nam; g Department of Biomedical Engineering, School of Medicine, Vietnam National University Ho Chi Minh City, Ho Chi Minh City 700000, Viet Nam

**Keywords:** SARS-CoV-2, COVID-19, Biomarker, CD24-CSF1R index, Immunity

## Abstract

Coronavirus disease 2019 (COVID-19), caused by SARS-CoV-2, has become one of the most serious public health crises worldwide. Most infected people are asymptomatic but are still able to spread the virus. People with mild or moderate illnesses are likely to recover without hospitalization, while critically ill patients face a higher risk of organ injury or even death. In this study, we aimed to identify a novel biomarker that can predict the severity of COVID-19 patients. Clinical information and RNA-seq data of leukocytes from whole blood samples with and without a COVID-19 diagnosis (n = 100 and 26, respectively) were retrieved from the National Center for Biotechnology Information Gene Expression Omnibus database. Raw data were processed using the Transcripts Per Million (TPM) method and then transformed using log_2_ (TPM+1) for normalization. The CD24-CSF1R index was established. Violin plots, Kaplan-Meier curves, ROC curves, and multivariate Cox proportional hazards regression analyses were performed to evaluate the prognostic value of the established index. The CD24-CSF1R index was significantly associated with ICU admission (n = 50 ICU, 50 non-ICU) and ventilatory status (n = 42 ventilation, 58 non-ventilation) with p = 4.186e-11 and p = 1.278e-07, respectively. The ROC curve produced a relatively accurate prediction of ICU admission with an AUC of 0.8524. Additionally, patients with a high index had significantly fewer mechanical ventilation-free days than patients with a low index (p = 6.07e−07). Furthermore, the established index showed a strong prognostic ability for the risk of using a ventilator in the multivariate Cox regression model (p < 0.001). The CD24-CSF1R index was significantly associated with COVID-19 severity. The established index could have potential implications for prognosis, disease severity stratification, and clinical management.

## 1 Introduction

The first known case of coronavirus disease 2019 (COVID-19) was identified in China in December 2019 [1]. It has now become an ongoing global pandemic. Common symptoms of COVID-19 include fever, cough, dyspnea, headache, sore throat, and runny nose [2]. There is no sign that the pandemic is going to be over anytime soon. Despite widespread vaccine acceptance, the COVID-19 situation remains challenging as new variants emerge. Consequently, the vaccine’s effectiveness remains limited [3,4].

There are several classification methods for COVID-19 patients. People who are infected with SARS-CoV-2 can be classified from having no symptoms to having critical illness based on their clinical manifestations, but the criteria for each category may overlap, and a patient’s clinical status may change over time with this classification approach [5]. SARS-CoV-2 viral loads were associated with worse respiratory disease severity, systemic inflammation, and an increased risk of death [6]. Interestingly, SARS-CoV-2 viral loads were found to be similar in asymptomatic and symptomatic patients [7]. Recently, smell dysfunction was used to identify infected cases at an early stage, but meaningful relationships between the test scores and disease severity were not found [8]. In addition, IL-6, C-reactive protein, and sIL6-R have been considered to be potential prognostic biomarkers for predicting critical illness and adverse outcomes [9,10]. While most infected people are in mild or moderate conditions and do not need hospitalization, the other 19% of patients develop severe symptoms and require assistance in the intensive care unit (ICU). Most of them also need some form of respiratory support [11]. Therefore, the ability to predict patients at high risk of progression will improve the prognosis, especially for those who need more care and intensive treatment.

The Casanova group has put forth a great effort in elucidating the genetic basis of severe COVID-19, which is mostly caused by insufficient interferon responses. Respiratory epithelial cells and plasmacytoid dendritic cells produce type I IFNs for host defense against SARS-CoV-2, in which insufficient type I IFN immunity during the first few days of illness may cause viral dissemination and critical pulmonary inflammation [12]. Besides, the cells or tissues that protect hosts against viral infection might suffer significant damage due to immune reactions [13]. Recent research showed a negative correlation between early cytokine increases and worse survival outcomes. Throughout the course of the disease, severe COVID-19 patients showed elevated responses in type 1 (antiviral), type 2 (anti-helminths), and type 3 (antifungal) responses compared to moderate ones [14]. Excessive, deleterious cytokine storms driven primarily by significant increases of IL-6/sIL-6R, IL-8, and IL-10 levels were also observed in severe COVID-19 patients compared with mild COVID-19 patients [10,15]. The findings implied that the immunopathology of COVID-19 was likely influenced by the abnormal activation of IFN-I signaling and a high level of inflammatory cytokines. Higher neutrophil counts were clinically seen in severe patients but not in moderate patients, which suggested that neutrophil overactivation may play a role in the progression of COVID-19 [13,16]. Additionally, natural killer (NK) cell immunotypes were recently reported to be related to the severity of COVID-19 disease [17]. These findings suggested that immune-related genes may serve as prognostic indicators for classifying COVID-19 patients.

*CD24* (small cell lung carcinoma cluster 4 antigen) plays an important role in modulating B-cell activation responses, which promote the antigen-dependent proliferation of B cells and prevent the differentiation of their terminals (nonpolymorphic regions) to form cell antibodies [18]. Its function is supposed to be a suppressor of antibody formation when SARS-CoV-2 affects the body. This is reinforced by the fact that MK-7110, a candidate drug targeting *CD24*, is currently being tested for COVID-19 patients in a phase III clinical trial by Merck. They reported that a single dose of MK-7110 displayed a 60% improvement in clinical status and decreased the mortality of infected patients by 50% compared to a placebo [19,20]. *CSF1R* (Colony Stimulating Factor 1 Receptor), primarily found in monocytes and macrophages, is a cell-surface receptor for *CSF1* and *IL34* [21]. It controls pro-inflammatory chemokines and plays an important role in the innate immune system and inflammatory development [22]. *CSF1R* is also involved in the regulation of hematopoietic precursor cell survival, proliferation, and differentiation [23]. In a recent study, a significant decrease in membrane *CSF1R* was observed in COVID-19 patients, suggesting that the inflammatory status of patients can be determined by *CSF1R* analysis. This could overcome the limitations of virology, bacteriology, and antibody assays [24].

In the present study, we aimed to establish a prognostic index to assist severity classification in COVID-19 patients. Two immune genes, *CD24* and *CSF1R*, which were significantly correlated with ICU admission and ventilatory status, were used to establish the gene expression difference, called the CD24-CSF1R index. The established index might be a novel predictor of COVID-19 severity.

## 2 Materials and methods

### 2.1 Data collection and reprocessing

RNA-seq datasets of COVID-19 patients were retrieved from the NCBI Gene Expression Omnibus database (https://www.ncbi.nlm.nih.gov/geo/). The data must contain information related to COVID-19 severity, such as admission into the ICU, disease severity, Charlson comorbidity index score, mechanical ventilatory status, age, and gender. Finally, we collected a blood-based RNA-seq dataset with accession number GSE157103 for our study [25]. It contained leukocyte samples from the blood plasma of 100 COVID-19 patients and 26 non-COVID-19 patients. All 126 patients had respiratory diseases and were admitted to Albany Medical Center in New York from April 6 to May 1, 2020. The raw RNA expression data were normalized using the Transcripts Per Million (TPM) method and then transformed using log_2_ (TPM + 1) [26,27]. A ROC curve analysis was conducted for each of the 19,000 genes to identify the candidates for predicting the severity of COVID-19 patients. Furthermore, *CD24* and *CSF1R* were chosen to predict the severity of COVID-19 patients based on a critical review of the roles and functions of the candidate genes. Normalized data were used to calculate the differential gene expression between *CD24* and *CSF1R* to establish the CD24-CSF1R index.

### 2.2 Correlation between the CD24-CSF1R index and COVID-19 severity

Normalized data were analyzed using ANOVA tests to compare the difference in the established index among ICU-admitted, non-ICU-admitted, ventilator-requiring, and non-ventilator-requiring COVID-19 patient groups. Then, a *t*-test was used to compare the difference in the established index between ICU-admitted and non-ICU-admitted patients. The analysis was also performed between ventilator-requiring and non-ventilator-requiring COVID-19 patient groups. The mRNA expression levels of *CD24* and *CSF1R* were also visualized. All analyses were represented as violin plots.

### 2.3 Evaluation of the ability of the established index to predict ICU admission in COVID-19 patients

To assess the performance of the CD24-CSF1R index, a ROC curve analysis was conducted using an online web tool named easyROC (http://www.biosoft.hacettepe.edu.tr/easyROC/) [28]. The file containing information about the ICU admission, CD24-CSF1R index, and Charlson score for each patient was uploaded to the website. ICU was set as the status variable, “yes” was set as the category for cases, and the ROC01 method was used to determine the optimal cut-off values for the next analysis.

### 2.4 Correlation between the CD24-CSF1R index and clinical information in COVID-19 patients

The COVID-19 patients were divided into two groups (high or low index) based on the cut-off value of the CD24-CSF1R index and Charlson score in the ROC analysis. The Kaplan-Meier curve analysis was performed to compare the mechanical ventilator-free days between these two groups. Univariate analysis was used to evaluate the correlation between the established index and clinical variables. Multivariate Cox proportional hazards regression analysis was performed to evaluate the impact of covariates including age, sex, and Charlson score on the independent prognostic ability of the CD24-CSF1R index.

### 2.5 Network analysis of protein interactions

The Search Tool for the Retrieval of Interacting Genes/Proteins (STRING) database v11.5 (http://www.stringdb.org/) was used to predict the protein interaction network of *CD24* and *CSF1R* with other proteins. The protein-protein interaction network was built based on publicly available sources of protein–protein interaction information and computational prediction methods. The analytical parameters were set according to the default indicators (i.e., network type: full STRING network; required score: medium confidence (0.400); FDR stringency: medium (5%)).

### 2.6 Statistical analysis

To assess the prognostic value of the established index, all statistical analyses were conducted using the R version 3.6.3, Python version 3.9.6 software (https://www.r-project.org/, https://www.python.org/) and their corresponding packages. T-tests were used to compare the difference between two groups of clustered data, while ANOVA tests were used to compare more than two groups, and the data were displayed as violin plots using the Seaborn Python package. Kaplan-Meier analysis was performed using the log-rank test from the survminer R package. The Chi-squared tests were used in the univariate analysis. All significance tests were two-sided, and a *p*-value of less than 0.05 was considered statistically significant.

## 3 Results

### 3.1 A high index score was significantly associated with COVID-19 severity

**Fig. 1:**
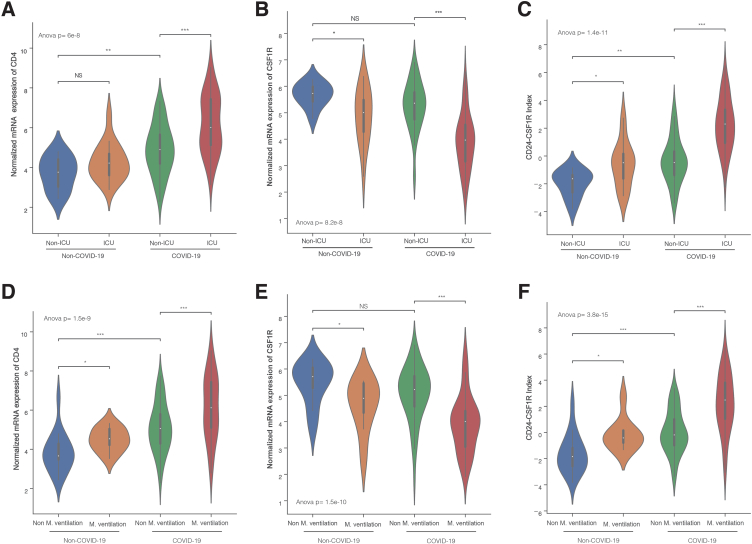
The violin plots of the mRNA expression level of *CD24*, *CSF1R*, and CD24-CSF1R index. Violin plots of the mRNA expression levels of *CD24* (A, D), *CSF1R* (B, E), and CD24-CSF1R index (C, F) between non-COVID-19 and COVID-19 patients with or without ICU admission and mechanical ventilation. Differences between two groups were estimated using *t*-test.

The normalized data were used to identify whether there was any difference in the mRNA expression levels of *CD24* and *CFS1R* between COVID-19 patients who were admitted to the ICU (n = 50) and those who were not admitted to the ICU (n = 50). The results showed that the mRNA expression level of *CD24* was significantly higher in patients with ICU settings (M = 6.174, SD = 1.473, Table 1) than in patients without ICU (M = 4.909, SD = 1.246, Table 1) (p = 1.102e-05, Fig. 1A). In contrast, *CSF1R* was highly expressed in the COVID-19/non-ICU group (M = 5.259, SD = 0.923, Table 1) compared to the COVID-19/ICU group (M = 3.921, SD = 1.062, Table 1) (p = 1.155e-09; Fig. 1B). Due to the distinct expression patterns between *CD24* and *CSF1R* in the context of ICU settings in COVID-19 patients, we integrated these two immune-related genes into an index so-called “CD24-CSF1R index”. When we used this established index, the violin plot indicated a significant difference between two groups of ICU-admitted (M = 2.253, SD = 1.884, Table 1) and non-ICU-admitted COVID-19 patients (M = −0.350, SD = 1.611, Table 1) (p = 4.186e-11, Fig. 1C).

**Table 1:** The mRNA expression level of CD24, CSF1R, and CD24-CSF1R index.

Gene marker	COVID-19 status	Intervention status	Count	Mean	SD	Min	25%	50%	75%	Max	IQR
CD24	Negative	Non-ICU	10	3.716	0.827	2.436	3.031	3.764	4.412	4.797	1.380
		ICU	16	4.279	0.925	2.889	3.610	4.221	4.691	6.661	1.081
	Positive	Non-ICU	50	4.909	1.246	2.281	4.190	4.902	5.658	7.340	1.468
		ICU	50	6.174	1.473	3.136	5.125	6.011	7.438	9.042	2.313
	Negative	Non-mechanical ventilation	17	3.839	0.990	2.436	3.465	3.660	4.282	6.661	0.817
		Mechanical ventilation	9	4.484	0.592	3.520	4.218	4.535	5.041	5.324	0.823
	Positive	Non-mechanical ventilation	58	5.102	1.252	2.281	4.286	5.058	5.798	7.745	1.513
		Mechanical ventilation	42	6.148	1.611	2.778	5.098	6.122	7.438	9.042	2.340
CSF1R	Negative	Non-ICU	10	5.679	0.448	4.779	5.423	5.730	5.990	6.259	0.567
		ICU	16	4.878	1.041	2.595	4.279	5.007	5.490	6.374	1.211
	Positive	Non-ICU	50	5.259	0.923	2.573	4.745	5.363	5.774	6.905	1.029
		ICU	50	3.921	1.062	1.761	3.156	3.967	4.550	6.548	1.394
	Negative	Non-mechanical ventilation	17	5.473	0.805	3.628	5.280	5.710	6.054	6.374	0.774
		Mechanical ventilation	9	4.644	0.975	2.595	4.340	4.888	5.460	5.544	1.120
	Positive	Non-mechanical ventilation	58	5.071	1.037	1.761	4.574	5.219	5.731	6.905	1.157
		Mechanical ventilation	42	3.925	1.087	1.907	3.046	4.005	4.403	6.548	1.357
CD24-CSF1R	Negative	Non-ICU	10	−1.964	0.939	−3.823	−2.625	−1.640	−1.365	−0.838	1.260
		ICU	16	−0.599	1.619	−2.866	−1.628	−0.486	0.161	2.730	1.789
	Positive	Non-ICU	50	−0.350	1.611	−3.396	−1.382	−0.473	0.328	3.631	1.710
		ICU	50	2.253	1.884	−2.217	0.934	2.298	3.340	6.499	2.406
	Negative	Non-mechanical ventilation	17	−1.634	1.463	−3.823	−2.589	−1.848	−1.199	2.244	1.391
		Mechanical ventilation	9	−0.161	1.210	−1.320	−0.789	−0.419	0.153	2.730	0.943
	Positive	Non-mechanical ventilation	58	0.031	1.646	−3.396	−0.989	−0.189	1.015	3.378	2.003
		Mechanical ventilation	42	2.223	2.203	−3.065	1.036	2.481	3.814	6.499	2.778

The mRNA expression levels of *CD24* and *CFS1R* were also used to evaluate their correlation with the COVID-19 patients' ventilatory status. The mRNA expression level of *CD24* was significantly upregulated in the COVID-19 patients who required ventilatory support (M = 6.148, SD = 1.611, Table 1) as compared to patients who did not need mechanical ventilation (M = 5.120, SD = 1.252, Table 1) (p = 4.175e-04) while the *CSF1R* exhibited an opposite trend (p = 5.859e-07) (Fig. 1D and E, and Table 1). In addition, the CD24-CSF1R index also showed a considerable distinction between the two groups in terms of ventilatory status (p = 1.278e-07; Fig. 1F, and Table 1). Taken together, these results indicate that the CD24-CSF1R index is significantly associated with COVID-19 severity.

### 3.2 The CD24-CSF1R index accurately classified the severity of COVID-19

**Fig. 2:**
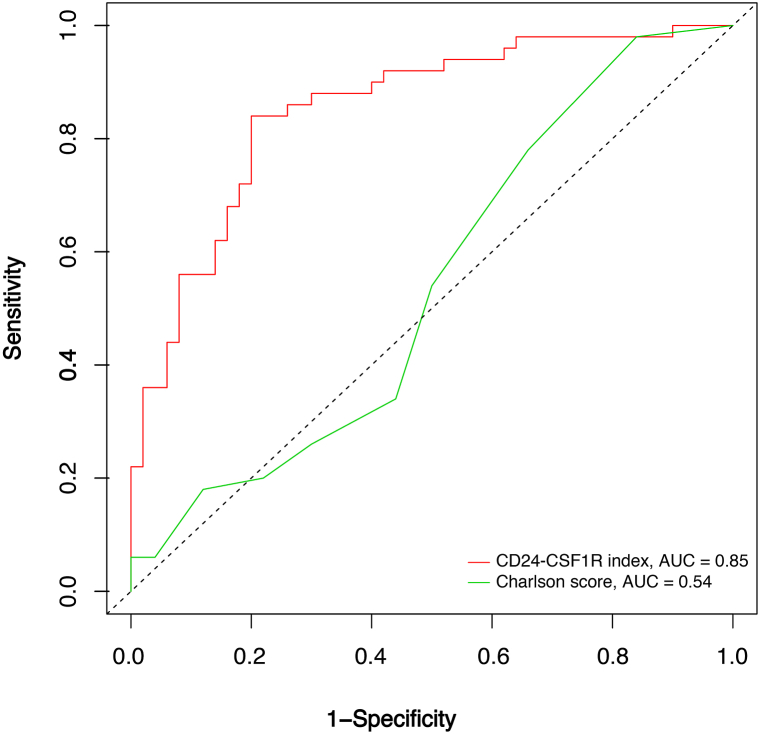
ROC curve analysis of the CD24-CSF1R index and Charlson score. The red line represents the established index, and the green line represents the Charlson score.

To identify how accurately the CD24-CSF1R index can classify COVID-19 patients, a web tool for ROC curve analysis was utilized to evaluate the ability of the established index to predict COVID-19 severity. Based on the information on ICU admission status, 100 COVID-19 patients were designated as a positive or negative class. The Area Under the Curve (AUC) is a useful summary of the Receiver Operating Characteristic (ROC) curve, which can be used to measure a classifier's ability to distinguish between groups. Specifically, the AUC indicates how efficiently a model is to predict accurately true positive and negative classes, in which the greater the AUC, the better the performance of the model. We identified an optimal cut-off index of 0.6923 as a potential inclusion standard for ICU admission of COVID-19 patients (AUC 0.8524, 95% CI 0.777–0.927). Sensitivity versus 1-specificity was plotted to build an ROC curve (Fig. 2). Interestingly, the model based on the CD24-CSF1R index showed a significantly better prediction of disease severity compared to the model based on the Charlson score (AUC 0.5402, 95% CI 0.426–0.655) [29]. The ROC curve produced a sensitivity of 84% (95% CI 71–93), a specificity of 80% (95% CI 66–90), a positive predictive value of 80.8% (95% CI 67–91), and a negative predictive value of 83.3% (95% CI 70–92) with this cut-off value. These performance measures appear to be superior to the Charlson score (Table 2). Therefore, the established index showed a better ability to classify COVID-19 patients at risk for ICU admission than the Charlson score.

**Table 2:** Performance Measures of the CD24-CSF1R index and Charlson score in ROC curve analysis.

Performance Measures	CD24	CSF1R	Charlson score	CD24-CSF1R Index
Cut-off value	5.1595	5.6212	3	0.6923
Sensitivity	0.74 (0.6–0.597)	0.12 (0.045–0.243)	0.54 (0.393–0.682)	0.84 (0.709–0.928)
Specificity	0.6 (0.74–0.452)	0.66 (0.512–0.788)	0.5 (0.355–0.645)	0.8 (0.663–0.9)
Positive Predictive Value	0.649 (0.698–0.504)	0.261 (0.16–0.454)	0.519 (0.373–0.663)	0.808 (0.674–0.912)
Negative Predictive Value	0.698 (0.649–0.545)	0.429 (0.207–0.589)	0.521 (0.375–0.664)	0.833 (0.699–0.918)
Positive Likelihood Ratio	1.85 (2.308–1.269)	0.353 (0.152–0.821)	1.08 (0.741–1.575)	4.2 (2.381–7.407)
Negative Likelihood Ratio	0.433 (0.541–0.258)	1.333 (1.066–1.668)	0.92 (0.611–1.384)	0.2 (0.104–0.383)

The values in the parentheses are the values within the 95% confidence interval.

### 3.3 The CD24-CSF1R index is an evaluable determinant for the requirement of mechanical ventilation in COVID-19 patients

**Fig. 3:**
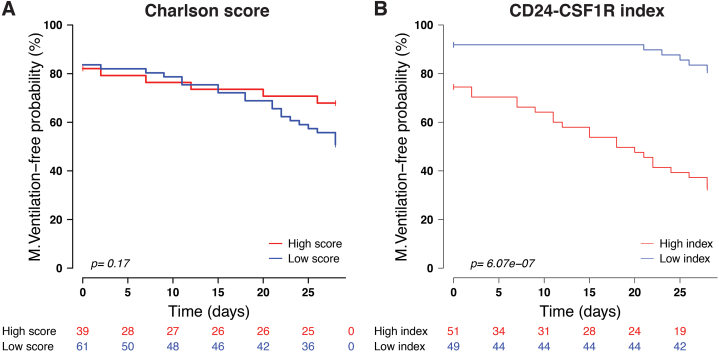
Kaplan-Meier curves of the CD24-CSF1R index and Charlson score based on ventilation status of COVID-19 patients. KM plots of Charlson score (A) and CD24-CSF1R index (B) of COVID-19 patients. Cut-off values of the CD24-CSF1R index and Charlson score from ROC curve analysis were used to divide COVID-19 patients into two risk groups (low index/score group versus high index/score group). The *p*-value was calculated by log-rank tests.

For further analysis, the COVID-19 patients were divided into two risk groups (low index group versus high index group) based on the above cut-off value of the CD24-CSF1R index. Kaplan-Meier curve analysis was performed on two groups of COVID-19 patients to confirm whether the established index was associated with ventilatory status. The cut-off value of the Charlson score was also used to divide COVID-19 patients into two groups (low score with≤3 versus high score with >3). The Kaplan-Meier curve was then conducted for comparison with the established index. The results showed that the Charlson score had no significant correlation with the ventilatory status of COVID-19 patients (p = 0.17, Fig. 3A). This implied that the Charlson score could not predict the risk of a patient requiring ventilatory support. In contrast, our established index is significantly associated with ventilatory support. In particular, patients with a high index had significantly fewer ventilator-free days than those with a low index (p = 6.07e−07, Fig. 3B).

### 3.4 The CD24-CSF1R index was found to be a reliable predictor of COVID-19 prognosis

**Fig. 4:**
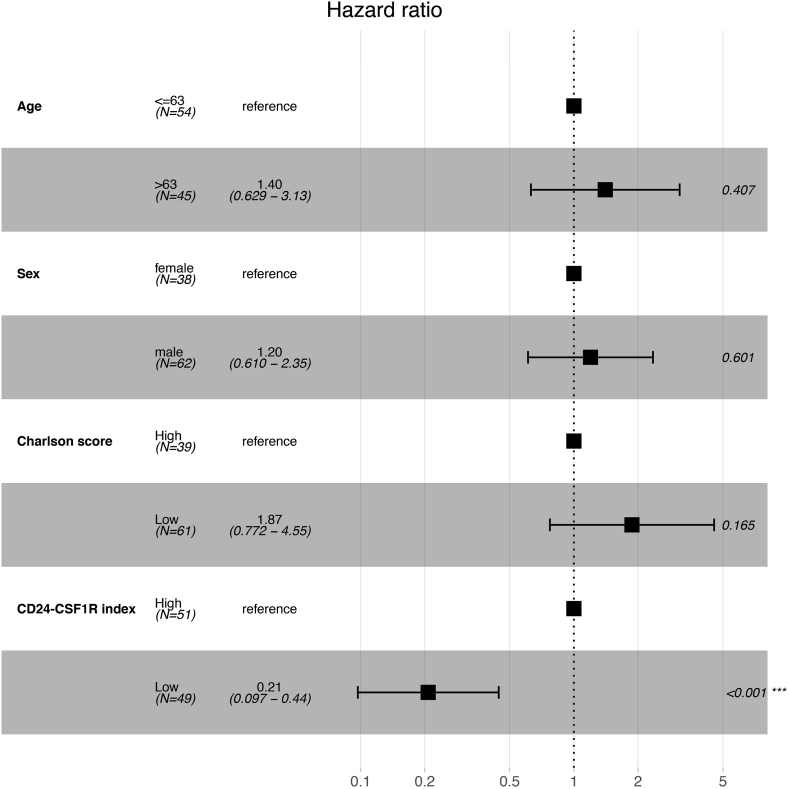
Multivariate Cox proportional hazards regression analysis of clinical variables. N is the number of COVID-19 patients in each group. The hazard ratio in brackets is the value within the 95% CI. The *p*-value was calculated based on the log-rank test and presented on the right outer side.

One of the most crucial analyses of the CD24-CSF1R index was its capacity to predict clinical outcomes. The established index was significantly associated with mechanical ventilation status and the Charlson score in univariate analysis (p < 0.005, Table 3). Multivariate Cox regression analysis was performed to confirm whether the CD24-CSF1R index could provide independent prognostic information for the mechanical ventilator requirement. Covariates, including age, sex, and Charlson score, were added to the model to evaluate factors that related to the independent prognostic ability of the established index. As shown in Fig. 4, the insignificant p-value results on the right outer side indicated that age, sex, and Charlson score showed no significant correlation with ventilatory support in COVID-19 patients, while the established index displayed statistical independence in predicting the risk of using a mechanical ventilator (p < 0.001). Furthermore, a hazard ratio of 5 demonstrated the index’s strong prognostic ability. In other words, if COVID-19 patients had a high CD24-CSF1R index recorded in their blood, there would be a five-time greater risk of requiring mechanical ventilator support than those with a low index. This suggests that the CD24-CSF1R index could be an independent predictor of the need for mechanical ventilation in COVID-19 patients.

**Table 3:** Clinical information of COVID-19 patients in two patient groups classified by the CD24-CSF1R index.

Variables		Total	*CD24-CSF1R index*		*p-*value
			High	Low	
Number of patients (%)		100	51 (51.00)	49 (49.00)	
**Age**	≤63	54 (54.00)	24 (24.00)	30 (30.00)	0.1323
	>63	45 (45.00)	27 (27.00)	18 (18.00)	
	NA	1 (1.00)	0 (0.00)	1 (1.00)	
**Sex**	female	38 (38.00)	19 (19.00)	19 (19.00)	1.00
	male	62 (62.00)	32 (32.00)	30 (30.00)	
**ICU admission**	no	50 (50.00)	10 (10.00)	40 (40.00)	5.49e-10
	yes	50 (50.00)	41 (41.00)	9 (9.00)	
**Machine ventilation**	no	50 (50.00)	18 (18.00)	40 (40.00)	2.69e-06
	yes	50 (50.00)	33 (33.00)	9 (9.00)	
**Charlson score**	0	9 (9.00)	2 (2.00)	7 (7.00)	0.012
	1	19 (19.00)	9 (9.00)	10 (10.00)	
	2	20 (20.00)	11 (11.00)	9 (9.00)	
	3	13 (13.00)	10 (10.00)	3 (3.00)	
	4	11 (11.00)	5 (5.00)	6 (6.00)	
	5	7 (7.00)	0 (0.00)	7 (7.00)	
	6	6 (6.00)	2 (2.00)	4 (4.00)	
	7	10 (10.00)	7 (7.00)	3 (3.00)	
	8	2 (2.00)	2 (2.00)	0 (0.00)	
	9	2 (2.00)	2 (2.00)	0 (0.00)	
	11	1 (1.00)	1 (1.00)	0 (0.00)	

Note: p -values were obtained from the χ2 -test.

### 3.5 Interactions and relationships of CD24 and CSF1R with other proteins

**Fig. 5:**
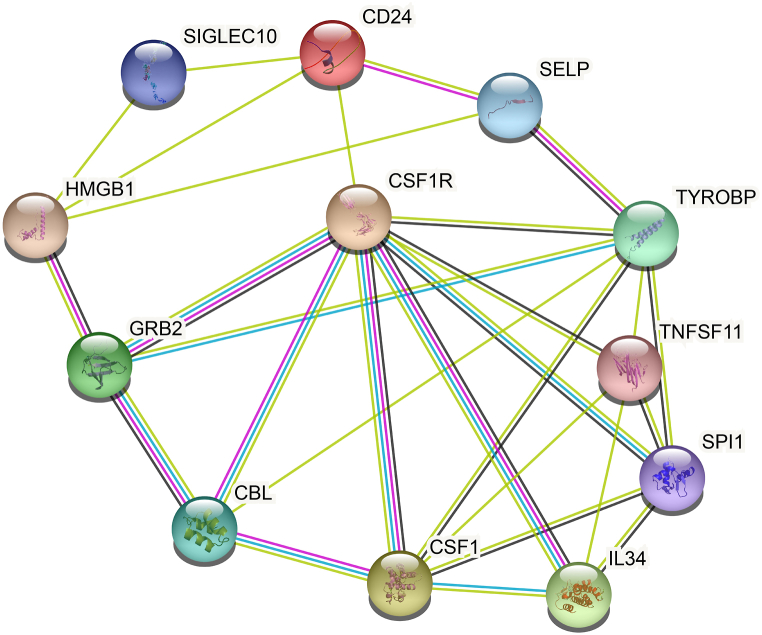
Protein interaction network of CD24 and CSF1R. This protein network was generated by STRING.

The protein–protein interaction network analysis was predicted using the STRING database v11.5. The analysis revealed an interactive network of *CD24* and *CSF1R* with ten other proteins, including sialic acid-binding Ig-like lectin 10 (SIGLEC10), P-selectin (SELP), high mobility group protein B1 (HMGB1), TYRO protein tyrosine kinase-binding protein (TYROBP), growth factor receptor-bound protein 2 (GRB2), tumor necrosis factor ligand superfamily member 11 (TNFSF11), transcription factor PU.1 (SPI1), interleukin-34 (IL34), macrophage colony-stimulating factor 1 (CSF1), E3 ubiquitin-protein ligase CBL (CBL) (Fig. 5). Among them, *CD24* was observed to interact with SELP, whereas *CSF1R* could interact with GRB2, CBL, and *CSF1*. *CSF1R* was also co-expressed with TYROBP, IL34, and SPI1.

## 4 Discussion

Although several vaccines are approved for human use, new coronavirus variants keep challenging the performance of current vaccines [30]. Additionally, adults who have been fully vaccinated can still carry the same viral load of coronavirus variants as unvaccinated people and may transmit the disease [31]. As a result, newly infected cases still emerge daily.

Acute respiratory disease syndrome (ARDS) was first observed in patients with severe COVID-19 through anatomical/histological reports from the lungs, which revealed excessive inflammatory activation and impairment of the bronchial and alveolar epithelium [32]. Recent studies have reported that lung and systemic host innate immune responses affect survival outcomes by triggering an uncontrolled inflammatory response called a “cytokine storm” [33]. The term “cytokine storm” refers to an activation cascade of auto-amplifying cytokine production caused by an out-of-control host immune response to various triggers [34]. These data suggested that immune-related genes may correlate with COVID-19 progression. Thus, these genes could be potential prognostic markers for classifying COVID-19 patients.

In our study, the mRNA expression level of *CD24* was seen to increase in critically ill patients (p = 1.102e-05, Fig. 1A). This was consistent with the results of a recently published study that found increased expression of CD24 to be strongly correlated with COVID-19 status and severity and involved in neutrophil degranulation. While *CD24* expression levels were higher in the severe patients than in the mild ones, the expression level of *CFS1R* showed an opposite expression pattern, which revealed a down-regulated expression in the severe patients (p = 1.155e−09, Fig. 1B). The up-regulation of *CD24* and down-regulation of *CSF1R* were an interesting observation. Thus, we decided to use them to establish the CD24-CSF1R index, in which a high-index score positively correlated with the requirement of mechanical ventilation and ICU admission. Specifically, COVID-19 patients with a high index had significantly shorter mechanical ventilator-free days and a higher risk of ICU admission than low-index ones. In the multivariate Cox proportional hazards regression analysis, the established index was found to be a strong independent biomarker that is self-reliable with other covariates. The CD24-CSF1R index’s classification ability was also superior to that of the Charlson score in the ROC analysis. Taken together, we suggest that the CD24-CSF1R index could be a novel tool to early classify and predict COVID-19 patients at high risk of progression.

Our protein interaction network analysis showed that CD24 and CSF1R interacted with multiple proteins that were involved in many immune signaling pathways such as macrophage colony-stimulating factor receptor binding, cytokine activity, cytokine receptor binding, and positive regulation of macrophage proliferation (Fig. 5). Macrophage colony-stimulating factor 1 (CSF1) regulates macrophage activation and immune responses. In many cases, CFS1 regulates the function of macrophages during viral infection, while respiratory viruses replicate within macrophages to escape immune detection [35]. The CSF1R requirement for the development of Langerhans cells (LCs) and microglia is interleukin 34 (IL34), which acts as a tissue-restricted ligand of CSF1R [36]. Mediators of multiple functions, LCs can interact directly with pathogens to produce effector cytokines and express different pattern recognition receptors to bind and capture pathogens [37]. Microglia are involved in phagocytosis and engulf various materials, such as cellular debris, lipids, invading viruses, and bacteria [38]. Moreover, CSF1 and SPI1 (transcription factor PU.1) are key regulators of pulmonary dendritic cells and macrophages [39]. Growth factor receptor-bound protein 2 (GBR2) is a receptor for interleukin-6 (IL6), whose serum levels significantly increase in COVID-19 patients [40]. Next, protein tyrosine kinase-binding protein (TYROBP) combined with killer-cell activating receptor-associated protein (KARAP) to form an adapter that is involved in a broad range of biological functions, such as anti-viral and anti-tumor activities [41], and displayed inflammatory reactions [42]. Sialic acid-binding Ig-like lectin 10 (SIGLEG10) expressed a large amount in B1 cells, which controls inflammation by producing interleukin-10 (IL-10), interleukin-35 (IL-35), and granulocyte-macrophage colony-stimulating factor (GM-CFS) [43]. Taken together, we found that our gene index interacts with various proteins that play important functions in immunity. This emphasizes the critical role of the established index in the progression of COVID-19 patients.

This study has several limitations. First, our findings should be validated in other independent datasets with a large number of patients in the control group. We could not find any available dataset with enough information to validate our results. Second, the CD24-CSF1R index should be validated by real-time PCR to demonstrate that the CD24-CSF1R index has the potential to predict COVID-19 severity.

In conclusion, the CD24-CSF1R index was significantly associated with COVID-19 severity and could have potential implications for prognosis, disease severity stratification, and clinical management. The index could be used as a molecular diagnostic test that may be performed along with the COVID-19 RT-PCR test to evaluate the severity of COVID-19 patients. Additionally, *CD24* and *CSF1R* may also potentially serve as novel targets for antiviral drug development, which effectively assists COVID-19 treatment.

## Ethics approval

Not applicable.

## Author contribution statement

Nguyen Thanh Đat: Conceived and designed the experiments; Performed the experiments; Analyzed and interpreted the data; Wrote the paper.

Nguyen Tan Thanh Giang: Conceived and designed the experiments; Performed the experiments; Analyzed and interpreted the data; Contributed reagents, materials, analysis tools or data.

Le Tam Vy: Performed the experiments; Analyzed and interpreted the data; Contributed reagents, materials, analysis tools or data; Wrote the paper.

Truong Ngoc Minh, Quynh Hoa Tran, Lam Thien Ngoc: Performed the experiments; Analyzed and interpreted the data; Wrote the paper.

Ngo Thanh Van: Performed the experiments; Analyzed and interpreted the data.

Nguyen Dinh Truong: Conceived and designed the experiments.

Minh Nam Nguyen: Conceived and designed the experiments; Contributed reagents, materials, analysis tools or data; Wrote the paper.

## Funding statement

This research did not receive any specific grant from funding agencies in the public, commercial, or not-for-profit sectors.

## Data availability statement

Data will be made available on request.

## Declaration of interest’s statement

The authors declare no competing interests.
